# Development and  initial validation of the Mindful Eating Behaviour Scale for children and parents

**DOI:** 10.1007/s40519-026-01881-1

**Published:** 2026-06-12

**Authors:** Natasha Dunsmore, Misba Hussain, Kyriaki Giannou, Michail Mantzios

**Affiliations:** 1 Department of Psychology, School of Health and Life Sciences, Birmingham City University, The Curzon Building, 4 Cardigan St, Birmingham, B4 7BD UK; 2 Department of Psychology, Faculty of Health and Life Sciences, De Montfort University, Leicester, UK

**Keywords:** Mindful Eating Behaviour Scale, Mindful eating behaviour, Mindful eating, Eating behaviours, Children’s eating behaviours, Children

## Abstract

**Purpose:**

This research aimed to address existing methodological limitations in research on mindful eating behaviours in children, to enhance future research in this area. This manuscript presents the development and the assessment of the validity and reliability of a Mindful Eating Behaviour Scale for children and parents across five studies.

**Methods:**

Study 1 assessed the scale's content validity using ratings from experts in the field. Study 2 evaluated the internal consistency of the scales. Study 3 explored temporal stability using a test–retest design with a 2-week interval. Studies 4 and 5 examined the criterion-related validity of the scales.

**Results:**

Study 1 demonstrated that the items reflected the definition of mindful eating behaviour and were suitable for children. Study 2 showed that the scale and the subscales, “*sensory attention*” and “*non-judgemental awareness*” demonstrated good internal consistency. Using exploratory factor analysis, the scale supported the presence of two factors, *“sensory attention” and “non-judgemental awareness”.* Study 3 showed that the scale was a reliable measure over time. Studies 4 and 5 showed that the scales are suitable measures for mindful eating behaviour. The scales demonstrated correlations between mindfulness and other eating behaviours, but further research is required, as only limited associations were observed.

**Conclusions:**

The current research presents new psychometric scales for mindful eating in children. The studies show that the scale is valid and reliable.

**Level of evidence:**

No level of evidence: basic science.

## Introduction

Over the last 50 years, the number of children and adolescents classified as overweight or obese has risen by 148 million; today, more than 390 million young people aged 5–19 are affected worldwide [1, 2]. Disordered eating behaviours affect approximately 22% of children; a figure that continues to rise, starting as young as 8 years old [3]. Such patterns are concerning for children’s health, as they increase the risk of developing associated health conditions, including cardiovascular diseases, malnutrition and eating disorders such as anorexia nervosa [4–10]. Preventing the escalation of disordered eating and obesity requires early identification of concerning behaviours like picky or emotional eating, and management through interventions [10, 11]. A promising strategy that directly assists children is the adoption of mindful eating [12–14]. Initial descriptions of mindful eating have been put forward, and subsequently refined into clearer, more scientifically informed definitions and measurement tools (e.g. mindful eating behaviour); tracing the development of theories and methods of measurement, the following section outlines a chronological review of the literature.

Typically, measuring mindful eating in children is through the Mindful Eating Questionnaire for Children (MEQ-C) [15], which aims to address problematic eating behaviours such as binge eating, emotional eating, and disordered eating, and promote healthier eating habits [15]. The MEQ-C was adapted from the adult Mindful Eating Questionnaire (MEQ) [16] and measures constructs such as portion control, eating in response to external cues, and eating while distracted. However, although related to mindful eating—for example, eating more mindfully may make someone more inclined to overcome distractions while eating—these do not directly reflect the concept of mindful eating behaviour [17–19]. Additionally, and importantly, the MEQ has been criticised for lacking reliability and validity. Zhang et al. [20] further highlighted the weaknesses of the MEQ, including inconsistent internal structure, poor internal reliability, and weak construct validity when used with an adolescent population. Interventions based on the MEQ-C have focused on teaching children strategies that correspond with the MEQ-C, such as portion control or emotion-based food choices, rather than simply on mindful eating behaviours [12, 21]. This means that such interventions, where the outcome was measured by changes in non-mindful eating behaviours, propose a methodological misalignment; that is, the studies cannot attribute their success to the cultivation of mindful eating or mindfulness, but rather to the taught and measured behavioural strategies, such as emotional eating. Such literature and method of measurement are not reflective of the true aspects of mindful eating behaviour, which is defined as “the sustained attention on a sensory element of the eating experience (e.g. the taste) and a non-judgemental (or non-evaluative) awareness of thoughts and feelings that are incongruent to the sensory elements of the present eating experience” [22].

To address such issues, the Mindful Eating Behaviour Scale-Trait (MEBS-T) was developed to accurately measure mindful eating behaviour in adults through the two key elements; *sensory attention* and *non-judgemental awareness* [17]. This scale aligns with the definition of mindful eating behaviour and has shown good reliability and validity in the adult version [17]. The goal of the present study was to develop a mindful eating behaviour scale, adapted from the adult version of the MEBS-T, that could be reliably administered to both children and parents. The inclusion of a parent‑report version of the scale is grounded in developmental theory and research on multi‑informant assessment in childhood [23, 24]. Parents observe eating-related behaviours that are at times less accessible to children’s introspection (e.g. responsiveness to food cues, self-regulation, emotional eating) [25]. While mindful eating contains internal components (e.g. sensory attention and non-judgmental awareness) that are best captured via child self‑report measures, it also comprises observable measures that parents are well placed to report. The parent version, therefore, provides a complementary perspective on the child’s eating behaviour.

### Present research

The present research consisted of five separate studies. The first study recruited experts in mindful eating, children, and eating behaviours to evaluate items from a newly developed psychometric scale. The experts were asked to assess whether the items reflected the definition of mindful eating and if they were appropriate for children. Based on these findings and the validation of the MEBS-T, a parental version of the scale was developed, assuming that relevance and clarity were ensured through previous and current experts of the validated items. The second study assessed the internal consistency and factorial structure of the scale whilst simultaneously assessing the parent version of the scale. The third study checked the reliability and stability of the scales over time, with participants completing the scales twice over a two-week interval. The fourth study examined the criterion-related validity of the children’s scale, and the final study examined the criterion-related validity of the parental scale. Studies four and five assessed whether the scale would be associated with other eating behaviours related to mindful eating. All studies received ethical approval. The sample size for each study was determined using guidelines from Cohen (1992) and suggestions from Devellis [26, 27].

## Methodology

### Study 1: Content Validity

#### Aim

To assess the content validity of the scale, examining whether it aligns with the definition of mindful eating and its suitability for a child population.

#### Participants

Fourteen expert participants from the UK were recruited directly via email for their research expertise. The eligibility criteria for the participants were to have expertise in either children, mindfulness, mindful eating or eating behaviours. The participants were all omnivores, with two reporting calorie restriction and one engaging in intermittent fasting. The scale includes questions about eating which could be deemed as a sensitive topic, therefore exclusion criteria included no mental health or eating disorder diagnosis. See Table 1 for all participant demographic information.

**Table 1:** Study 1 participant demographics

Demographics	*n*
Gender	
Female	12
Male	2
Ethnicity	
White	13
Black	1
Profession	
Professor	2
University lecturer	5
Researcher	4
Dietician	2
Clinician	1
Area of expertise	
Nutrition and/or eating behaviours	8
Mindfulness	3
Mindful eating	1
Children	2
Diet	
Omnivores	14

*N* = 14. Participants were on average 36.6 years old (SD = 9.45)

#### Materials

##### Mindful Eating Behaviour Scale for Children (MEBS-C)

The Mindful Eating Behaviour Scale for Children (MEBS-C) is an 8-item self-report scale that measures a child’s mindful eating behaviour. This scale was adapted from the adult version of the Mindful Eating Behaviour Scale-Trait (MEBS-T) by the last author, Mantzios [17]. A period of consultation occurred with co-authors and developmental psychologists specialising in children and language to finalise the version to be tested. The scale and items were assessed for readability using the Flesch reading ease and the Flesch–Kincaid grade tests [28]. Such tests ensured that the right target group would be exposed to the scale by assessing comprehension difficulty and providing an estimate of educational grade level.

Findings (Flesch–Kincaid Grade Level: 1.3, Flesch Reading Ease Score: 98.1) indicated that the measure could be easily understood by children as young as 5 years old [28]. Nevertheless, the potential limitations of such tests [29] and the potential need for later validation with scales that were developed with specific age groups required a higher age range for criterion-related and concurrent validity. Also, the executive functioning and depth of reflection observed in items assessing self-regulation while eating may indicate that children over the age of 7 may be more suitable to comprehend these items fully [30, 31]. Therefore, we assessed the reliability and validity of the scale in the age range of 8–12 years.

In line with the definitions and theory of mindful eating, the MEBS-C includes two subscales that measure key aspects of mindful eating, “Sensory Attention” (the sensory experience of food, e.g. the taste) and “Non-judgemental Awareness” (adopting a non-judgemental or non-evaluative awareness of thoughts and feelings that are unrelated to the eating experience) [17]. Items 1–4 reflect “Sensory Attention”, such as “I really taste and enjoy my food.” Items 5–8 reflect “Non-judgemental Awareness”, such as "when I’m eating, and I have thoughts and feelings, I stay focused on the food.” Responses are recorded using a 4-point Likert scale, ranging from 1 (I really disagree) to 4 (I really agree). See Table 2 for item comparison between MEBS-T, MEBS-C and the Mindful Eating Behaviour Scale for Parents (MEBS-P).

**Table 2:** Items of MEBS-T, MEBS-C and MEBS-P

Items	MEBS-T	MEBS-C	MEBS-P
1	I fully taste what I am eating	I really taste and enjoy my food	My child fully tastes what they are eating
2	I notice the smell, texture and/or colours of the food I am eating	I pay attention to how my food smells, feels, and looks	My child notices the smell, texture and/or colours of the food they are eating
3	I focus on what I am eating	I focus on what I’m eating	My child focuses on what they are eating
4	I fully taste every bite that I am eating	I make sure to taste every bite of my food	My child fully tastes every bite that they are eating
5	I notice thoughts and/or feelings that are unrelated to my eating, but I redirect my attention to the food and the experience of eating	When I have thoughts and feelings that have nothing to do with eating, I bring my attention back to the food and eating	My child notices thoughts and/or feelings that are unrelated to their eating but redirects their attention to the food and the experience of eating
6	When I am eating, I have thoughts and/or feelings but keep refocusing on the food	When I’m eating and I have thoughts and feelings, I stay focused on the food	When my child is eating, they have thoughts and/or feelings but keep refocusing on the food
7	I hold my attention on what I am eating, despite recognising the occurrence of thoughts and/or feelings while I am eating	I stay focused on my food even when other thoughts and feelings pop up	My child holds their attention on what they are eating despite recognising the occurrence of thoughts and/or feelings while eating
8	When I am eating, I overcome unrelated thoughts and/or feelings by focusing on the food and the sensation of eating	I think about eating and how good the food is and do not spend time with other thoughts and feelings	When my child is eating, they overcome unrelated thoughts and/or feelings by focusing on the food and the sensation of eating

### Procedure

Participants were recruited from the University via email. Once invited to the study, participants were asked to read the information sheet and complete the consent form to continue. Participants were then presented with demographic questions about age, gender, ethnicity, diet, and profession. Next, participants were provided with the same definition of mindful eating behaviour as presented in the introduction to this manuscript. Using this definition, participants assessed the items of the MEBS-C. For each item, they would answer the following two questions: 1. “How relevant is this to the definition of mindful eating provided?” 2. “How suitable is this item for children aged 8–12?” The responses were recorded using a Likert scale, from 1 (very poor) to 5 (very good).

#### Data analysis

Aiken’s *V* formula was used to analyse the content validity of the scale, referring to the definition of mindful eating and its appropriateness for children.

#### Results

For the definition of mindful eating, the scale demonstrated good content validity, with an overall Aiken’s *V* value of 0.94. The “Sensory Attention” subscale produced a *V* value of 0.93, and the “Non-judgemental Awareness” subscale produced 0.95. These high values indicate a strong agreement among expert raters that the scale and its subscales accurately reflect the definition of mindful eating.

Regarding the scale’s suitability for use with children, the overall Aiken’s *V* was 0.85, with “Sensory Attention” scoring 0.90 and “Non-judgemental Awareness” scoring 0.80. These high values suggest that the scale is considered appropriate for use with child populations. See Table 3 for all statistics.

**Table 3:** Aiken’s *V* values obtained from expert reviewers’ ratings for Mindful Eating Behaviour Scale for Children

	Expert evaluation	
	Relevance to the defintion of mindful eating	Suitability for children
Item 1	0.91	0.91
Item 2	1	0.96
Item 3	0.93	0.89
Item 4	0.88	0.82
Item 5	0.96	0.77
Item 6	0.95	0.84
Item 7	0.95	0.84
Item 8	0.95	0.77

#### Summary of findings

Study 1 produced high Aikens *V* values, showing that the scale items accurately reflect the definition of mindful eating behaviours as well as being suitable for child populations. These findings indicate the scales have high content validity.

### Study 2: Internal Consistency

#### Aim

To assess the internal consistency of the scale, by examining whether the items on the scale measure mindful eating, and whether the scale displays two separate factors, using exploratory factor analysis.

#### Participants

Three-hundred and forty-one UK parent–child pairs were recruited through Prolific; an online recruitment programme used for research. Participants gave demographic information about their children, including age, gender, ethnicity diet and picky eating behaviour or any sensory issues surrounding food. Collecting this information allows exploration of the sample as well as replication in future research. Exclusion criteria included no mental health or eating disorder diagnosis. Eligibility criteria required the children to be between aged 8–12 years old. See Table 4 for all participant demographics.

**Table 4:** Study 2 participant demographics

Demographics	*n*
Gender	
Male	144
Female	148
Non-binary	3
Unknown	46
Ethnicity	
White	246
Asian	14
Black	15
Mixed race	19
Unknown	47
Diet	
Omnivore	188
Occasional omnivore	12
Lacto-ovo-vegetarian	3
Semi-vegetarian	5
Lactovegetarian	1
Unknown	132
Picky eaters	112

*N* = 341. Participants were on average age 9.7 years, 138.2 cm in height and 35.1 kg in weight. Average BMI was 19

#### Materials

##### The Mindful Eating Behaviour Scale for Children (MEBS-C)

Please refer to study 1 materials for a full description of the scale. The scale demonstrated good internal consistency (*α* = 0.83), and so did each subscale, “Sensory Attention” (*α* = 0.68), and “Non-judgemental Awareness” (*α* = 0.85).

##### The Mindful Eating Behaviour Scale for Parents (MEBS-P)

The Mindful Eating Behaviour Scale for Parents (MEBS-P) is an 8-item scale designed to assess how parents perceive their child’s mindful eating behaviour. All items align with the definition and theory of mindful eating behaviour, and the scale was adapted by the last author, Mantzios [17], from the adult version, and the child version of the mindful eating behaviour scale (MEBS-T & MEBS-C). The scale includes two subscales. Items 1–4 focus on the “Sensory Attention” aspect of mindful eating. An example of a sensory attention item is: "My child notices the smell, texture, and colours of the food they are eating." Items 5–8 relate to the “Non-judgmental Awareness” aspect. An example of this is: "When my child is eating, they have thoughts and feelings but keep refocusing on the food." Parents respond using a 4-point Likert scale, ranging from 1 (strongly disagree) to 4 (strongly agree). The scale showed good internal consistency (*α* = 0.85), as well as the subscales, “Sensory Attention” (*α* = 0.75), and “Non-judgemental Awareness” (*α* = 0.86).

#### Procedure

Most of the parents and children were invited to participate through Prolific, where they were compensated for their time (£6.00/h). Some of the participants were recruited through a school and others through social media platforms (LinkedIn and Facebook), but this was minimal (*N* = 14). Once parents had read the information sheet and completed the consent form, they filled out demographic information about their child. This included age, gender, ethnicity, height, weight, and some questions about the child’s diet. Parent participants then moved on to the MEBS-P, where they answered questions about their child’s mindful eating behaviour. Once completed, parent participants were prompted to hand over their device to their child to allow their child to answer the MEBS-C. Before completing, the children read a brief description about the study, via a child-friendly information sheet simply outlining that they will be asked some questions about eating, and gave their consent. They then completed the MEBS-C, and then a debrief form was presented for the parent to read.

#### Data analysis

A Kaiser–Meyer–Olkin (KMO) measure of sampling adequacy was performed to check if the data were suitable for factor analysis. An Exploratory Factor Analysis (EFA) was conducted on both scales to assess how accurate the items measured mindful eating behaviour and whether they reflected the intended subscales, “Sensory Attention”, and “Non-judgemental Awareness”. All data were analysed using IBM SPSS 30.

#### Results

##### MEBS-C

Exploratory factor analysis was performed on all 8 items of the MEBS-C. A KMO value of 0.83 and Bartlett’s test of sphericity (*p* < 0.001) proved that factor analysis was suitable for the present data. Principal component analysis identified two components with eigenvalues > 1, accounting for 46.4% and 14.9% of the variance. Oblique rotation was performed, based on the assumption that the subscales would be correlated, as all the items were designed to measure mindful eating behaviour. The two components explained 61.3% of the total variance. Factor analysis showed strong loadings (> 0.67). The correlation between the subscales was moderately strong (*r* = 0.42), which is expected, as overall the items are measuring the same underlying construct. See Table 5 for exploratory factor analysis statistics for MEBS-C.

**Table 5:** Factor loadings for exploratory factor analysis with oblique rotation of Mindful Eating Behaviour Scale for Children

	1	2
Item 1		0.721
Item 2		0.667
Item 3		0.672
Item 4		0.693
Item 5	0.842	
Item 6	0.886	
Item 7	0.867	
Item 8	0.713	

##### MEBS-P

Exploratory factor analysis was performed on all 8 items of the MEBS-P. A KMO value of 0.85 and Bartlett’s test of sphericity (*p* < 0.001) proved that factor analysis was suitable for the present data. Principal component analysis identified two components with eigenvalues > 1, accounting for 48.9% of and 17% the variance. Oblique rotation was performed, based on the assumption that the subscales would be correlated, as all the items were designed to measure mindful eating behaviour. The two components explained 68.9% of the total variance. Factor analysis showed strong loadings (> 0.78), apart from item 4 (0.45). The correlation between the subscales was moderately strong (*r* = 0.41), which is expected, as overall the items are measuring the same underlying construct. See Table 6 for exploratory factor analysis statistics for MEBS-P.

**Table 6:** Factor loadings for exploratory analysis with oblique rotation of Mindful Eating Behaviour Scale for Parents

	1	2
Item 1		0.811
Item 2		0.815
Item 3		0.777
Item 4		0.451
Item 5	0.785	
Item 6	0.834	
Item 7	0.839	
Item 8	0.891	

#### Summary of findings

Study 2 showed that both scales produced good internal consistency, as well as each subscale. Exploratory factor analysis showed that the scales presented two factors, which are “Sensory Attention” and “Non-judgemental Awareness”.

### Study 3: Test–retest Reliability

#### Aim

To assess test–retest reliability, observing the consistency of the results over a 2-week period.

#### Participants

One-hundred and four UK parent–child pairs were recruited through Prolific. Prior to releasing the study, screeners were added to exclude participants that had already taken part in study 2. Participants gave demographic information about their children, including age, gender, ethnicity diet and picky eating behaviour or any sensory issues surrounding food. Other exclusion criteria included no history of a mental health or eating disorder diagnosis. Eligibility required children to be between 8 and 12 years of age. See Table 7 for all participant demographics.

**Table 7:** Study 3 participant demographics

Demographics	*n*
Gender	
Male	58
Female	46
Ethnicity	
White	90
Asian	7
Mixed race	5
Black	1
Picky eaters	37

*N* = 104. Participants were on average age 9.7 years, 139.9 cm in height and 35.1 kg in weight. Average BMI was 18.2

#### Materials

##### The Mindful Eating Behaviour Scale for Children (MEBS-C).

Please refer to study 1 materials for a description of the MEBS-C. In the present study, the scale showed good internal consistency initially (*a* = 0.86) and on retest (*a* = 0.91). The subscales also demonstrated strong internal consistency, with “Sensory Attention” showing Cronbach’s alpha values of 0.71 initially and 0.82 at re-test, and “Non-judgemental Awareness” showing values of 0.9 initially and 0.93 at re-test.

##### The Mindful Eating Behaviour Scale for Parents (MEBS-P)

Please refer to study 2 materials for a description of the MEBS-P. For the present study, the scale showed good internal consistency initially (*a* = 0.86) and in the re-test (*a* = 0.87). Good internal consistency was also seen in the subscales, “Sensory Attention” (*a* = 0.84 initially, *a* = 0.86 at re-test) and “Non-judgemental Awareness” (*a* = 0.87 initially,* a* = 0.88 at re-test).

#### Procedure

Parents were invited to participate using Prolific, where they were compensated for their time (£6.00/h). Once parents had read the information sheet and consented for themselves and their child to participate, they answered some demographic questions about their child. They were then directed to the MEBS-P. Once completed, parents were asked to pass their device to their child, who would read a child-friendly information sheet and provide assent to participate. The child participant would then complete the MEBS-C. This would then be followed by a debrief form for the parent to read. Two weeks later, the same participants were re-invited via Prolific to take part in the follow-up study, during which the same procedure was repeated. Participants were reimbursed for the second study (£7.09/h).

#### Data analysis

A Pearson's correlation was conducted for both scales to assess the test–retest reliability between test 1 and test 2. All data were analysed using IBM SPSS 30.

#### Results

##### MEBS-C

A Pearson’s correlation revealed a strong positive correlation for the total score of the scale (*r* = 0.79, *p* < 0.001), suggesting very good test–retest reliability for the whole scale. “Sensory Attention” (*r* = 0.64,* p* < 0.001) and “Non-judgemental Awareness” (*r* = 0.58, *p* < 0.001) had moderate to strong positive correlations between test 1 and 2, indicating good test–retest reliability within the subscales.

##### MEBS-P

A Pearson’s test showed a moderate to strong positive correlation (*r* = 0.62, *p* < 0.001) between test 1 and test 2, suggesting that the scale has good test–retest reliability. “Sensory Attention” (*r* = 0.65, *p* < 0.001) and “Non-judgemental Awareness” (*r* = 0.52, *p* < 0.001) subscales also demonstrated a moderate to strong positive correlation and good test–retest reliability between test 1 and test 2.

#### Summary of findings

Findings from study 3 indicate that the scales demonstrate good test–rest reliability over time. Specifically, scores obtained at the initial assessment were strongly correlated with those collected after the two-week interval.

### Study 4: Criterion-related Validity 1 (child only)

#### Aim

Assessing criterion-related validity, whether the MEBS-C shows how mindful eating behaviours can predict other eating behaviours in children.

#### Participants

A total of 137 UK child participants were recruited through their parents/guardians using Prolific. Participants gave demographic information about their children, including age, gender, ethnicity diet and picky eating behaviour or any sensory issues surrounding food. Exclusion criteria included no mental health or eating disorder diagnosis. Eligibility required children to be between 8 and 12 years of age. See Table 8 for all participant demographics.

**Table 8:** Study 4 participant demographics

Demographics	*n*
Gender	
Male	67
Female	70
Ethnicity	
White	114
Asian	5
Mixed race	12
Black	6
Diet	
Omnivore	126
Occasional omnivore	7
Semi-vegetarian	2
Lacto-ovo-vegetarian	2
Picky eaters	46

*N* = 137. Participants were on average age 9.5 years, 136 cm in height and 33.9 kg in weight. Average BMI was 18.1

#### Materials

##### The Mindful Eating Behaviour Scale for Children (MEBS-C)

Please refer to study 1 materials to obtain a complete description of the MEBS-C. For the current study, the MEBS-C (*a* = 0.79) and the subscales “Sensory Attention” (*a* = 0.63) and “Non-judgemental Awareness” (*a* = 0.79) displayed good internal consistency.

##### Dutch Eating Behaviour Questionnaire for Children (DEBQ-C) [32]

The DEBQ-C is a 20-item questionnaire used to measure emerging dietary restraint and overeating behaviours in young children [32]. It is a self-report measure assessing three types of eating behaviours in children, made up by three subscales; “Restrained Eating”, “Emotional Eating”, and “External Eating” [32]. Sample questions from this scale include “Do you watch what you eat?” “Do you desire to eat when you feel lonely?” “Are you tempted by delicious foods?” [32]. Children answer by selecting either “no”, “sometimes” or “yes” [32]. The DEBQ-C (*a* = 0.78) displayed good internal consistency. “Emotional Eating” (*a* = 0.88) and “External Eating” (*a* = 0.70) also displayed good internal consistency. “Restrained Eating” (*a* = 0.56) displayed moderate internal consistency.

##### Kids-Palatable Eating Motives Scale (K-PEMS) [33]

The K-PEMS is a scale that assesses the different reasons behind children eating calorie-dense foods [33]. It is a 19-item self-report scale that includes four subscales: “Coping Motive”, “Reward Enhancement Motive”, “Social Motive”, and “Conformity Motive” [33]. Children are presented with a list of “tasty food and drink”, such as ice-cream, soda, chips, pizza, and cookies [33]. The children are asked to reflect on times when they consume foods like these, and whether it is due to specific reasons [33]. For example, reasons included are “I eat these foods to forget things that I am worrying about”, “I eat these foods because it is exciting”, “I eat these foods to enjoy time with friends” [33]. Children answer using a 5-point Likert scale, ranging from “Never/Almost never” to “Almost always/Always” [33]. K-PEMS (*a* = 0.92) and all its subscales, “Coping Motive” (*a* = 0.83), “Reward Enhancement Motive” (*a* = 0.85), “Social Motive” (*a* = 0.88) and “Conformity Motive” (*a* = 0.85) all displayed good internal consistency.

##### Child and Adolescent Mindfulness Measure (CAMM) [34]

CAMM is a 10-item self-report measure of mindfulness behaviours in children [34]. It is a single-factor scale, with higher scores indicating higher levels of mindfulness engagement [34]. Sample items from the scale include “I get upset with myself for having feelings that don’t make sense” and “I push away thoughts I don’t like” [34]. Scoring is on a 5-point Likert scale from 0 (Never true) to 4 (Always true) [34]. CAMM (*a* = 0.77) demonstrated good internal consistency.

#### Procedure

Child participants were recruited through parents via Prolific and were compensated for their time (£6.00/h). After reading the information sheet and consenting, parents filled out the demographic information about their child. Parents were then presented with a supporting document for the scales, with tips on helping their child through the study, if needed. Child participants were then given the device and they read a child information sheet and provided assent before moving onto the study. Children then completed the MEBS-C, DEBQ-C, K-PEMS and CAMM. Following the study, a debrief form was presented for participants to read.

#### Data analysis

A Pearson’s correlation was performed between the MEBS-C and each scale, and between the subscales to determine associations between mindful eating behaviours and other eating behaviours. To consider individual differences between male and female participants within this age range, and to enhance the understanding of the data, further exploratory analyses were conducted and gender was controlled for. All data were analysed using IBM SPSS 30.

#### Results

For all participants there was a significant, weak positive correlation between “Sensory Attention” and “Restrained Eating” (*r* = 0.26, *p* = 0.006), and between “Non-judgemental Awareness and “Conformity Motive” (*r* = 0.3, *p* = 0.04) (see Table 9).

**Table 9:** Pearson’s correlations were conducted between the MEBS-C, DEBQ-C, CAMM and K-PEMS for all participants

	1	2	3	4
1. MEBS-C (SA)	1			
2. MEBS-C (NJA)	0.500**	1		
3. DEBQ-C (RE)	0.258**	0.038	1	
4. K-PEMS (CFM)	0.141	0.300**	0.206*	1

*N* = 137

(1) MEBS-C (SA) = *The Mindful Eating Behaviour Scale for Children,* Sensory Attention, (2) MEBS-C (NJA) = *The Mindful Eating Behaviour Scale for Children,* Non-judgemental Awareness, (3) DEBQ-C (RE) = *Children’s Dutch Eating Behaviour Scale,* Restrained Eating, (4) K-PEMS (CFM) = *Kids-Palatable Eating Motives Scale,* Conformity Motive

*Correlation is significant at the 0.05

^**^Correlation is significant at the 0.01

Female participants displayed a significant, moderately negative correlation between MEBS-C and CAMM total scores (*r* =—0.32, *p* = 0.02). There was a significant moderate positive correlation between “Sensory Attention”, “Restrained Eating” (*r* = 0.32, *p* = 0.02) and a significant, moderately negative correlation between “Non-judgemental Awareness” and CAMM total score (*r* = -0.34, *p* = 0.009) (see Table 10).

**Table 10:** Pearson’s correlations were conducted between the MEBS-C, DEBQ-C and CAMM for female participants

	1	2	3	4	5
1. MEBS-C	1				
2. MEBS-C (SA)	0.838**	1			
3. MEBS-C (NJA)	0.871**	0.461**	1		
4. DEBQ-C (RE)	0.201	0.324*	0.088	1	
5. CAMM	− 320*	− 0.228	− 0.341**	− 0.343*	1

*N* = 137

(1) MEBS-C = *The Mindful Eating Behaviour Scale for Children,* (2) MEBS-C (SA) = *The Mindful Eating Behaviour Scale,* Sensory Attention, (3) MEBS-C (NJA) = *The Mindful Eating Behaviour Scale,* Non-judgemental Awareness, (4) DEBQ-C (RE) = *Dutch Eating Behaviour Questionnaire for Children,* Restrained Eating, 5. CAMM = *Child and Adolescent Mindfulness Measure*

*Correlation is significant at the 0.05

^**^ Correlation is significant at the 0.01

There were many significant correlations for male participants. There were significant, moderately positive correlations between MEBS-C total score and “Emotional Eating” (*r* = 0.28, *p* = 0.04), K-PEMS total score (*r* = 0.44, *p* = 0.002), “Coping Motive” (*r* = 0.39, *p* = 0.006), “Social Motive” (*r* = 0.32,* p* = 0.03) and “Reward Enhancement Motive” (*r* = 0.44,* p* = 0.002). There were significant, moderately positive correlations between “Sensory Attention” subscale and K-PEMS total score (*r* = 0.41,* p* = 0.003), “Coping Motive” (*r* = 0.39, *p* = 0.004), “Social Motive” (*r* = 0.31, *p* = 0.03), and “Reward Enhancement Motive” (*r* = 0.44, *p* < 0.001). There were significant, moderately positive correlations between “Non-judgemental Awareness” subscale and K-PEMS total score (*r* = 0.35, *p* = 0.01), “Coping Motive” (*r* = 0.31, *p* = 0.03) and “Reward Enhancement Motive” (*r* = 0.3, *p* = 0.04) (see Table 11). There were no significant relationships between K-PEMS scores and MEBS-C scores in female participants.

**Table 11:** Pearson’s correlations were conducted between the MEBS-C, DEBQ-C, CAMM and K-PEMS for male participants

	1	2	3	4	5	6	7	8
1. MEBS-C	1							
2. MEBS-C (SA)	0.846**	1						
3. MEBS-C (NJA)	0.902**	0.532**	1					
4. DEBQ-C (EE)	0.281*	0.216	0.221	1				
5. K-PEMS	0.436**	0.410**	0.351*	0.512**	1			
6. K-PEMS (CM)	0.393**	0.392*	0.306*	0.676**	0.764**	1		
7. K-PEMS (SM)	0.318*	0.305*	0.263	0.360*	0.853**	0.468**	1	
8. K-PEMS (REM)	0.441**	0.444**	0.295*	0.387**	0.742**	0.446**	0.534**	1

*N* = 137

*N* = 137

(1) MEBS-C = *The Mindful Eating Behaviour Scale for Children,* (2) MEBS-C (SA) = *The Mindful Eating Behaviour Scale for Children,* Sensory Attention, (3) MEBS-C (NJA) = *The Mindful Eating Behaviour Scale for Children,* Non-judgemental Awareness, (4) DEBQ-C (EE) = *Dutch Eating Behaviour Questionnaire for Children,* Emotional Eating, (5) K-PEMS = *Kids-Palatable Eating Motives Scale,* (6) K-PEMS (CM) = *Kids-Palatable Eating Motives Scale,* Coping Motive, (7) K-PEMS (SM) = *Kids-Palatable Eating Motives Scale,* Social Motive, (8) K-PEMS (REM) = *Kids-Palatable Eating Motives Scale,* Reward Enhancement Motive

*Correlation is significant at the 0.05

^**^ Correlation is significant at the 0.01

### Study 5: Criterion-related Validity 2 (parent only)

#### Aim

Assessing criterion-related validity, whether the MEBS-P shows how mindful eating behaviours can predict other eating behaviours in children.

#### Participants

Ninety-three UK parent participants were recruited using Prolific. It is important to note that these parent participants are not the parents of the study 4 participants. Participants gave demographic information about their children, including age, gender, ethnicity diet and picky eating behaviour or any sensory issues surrounding food. Exclusion criteria included no mental health or eating disorder diagnosis. Eligibility required participant’s children to be between 8 and 12 years of age. See Table 12 for all participant demographics.

**Table 12:** Study 5 participant demographics

Demographics	*n*
Gender	
Male	53
Female	40
Ethnicity	
White	72
Asian	6
Mixed race	7
Black	6
Unknown	2
Diet	
Omnivore	86
Occasional omnivore	3
Semi-vegetarian	2
Lacto-ovo-vegetarian	1
Vegan	1
Picky eaters	30

*N* = 93. Participants were on average age 9.5 years, 138 cm in height and 35.1 kg in weight. Average BMI was 18.6

#### Materials

##### The Mindful Eating Behaviour Scale for Parents (MEBS-P)

Please see study 2 materials for a full description of the scale. For the present study, the scale demonstrated good internal consistency (*a* = 0.84). The subscales, “Sensory Attention” (*a* = 0.8) and “Non-judgemental Awareness” (*a* = 0.84) also showed good internal consistency.

##### Children’s Eating Behaviour Questionnaire (CEBQ) [25]

The Children’s Eating Behaviour Questionnaire is a 35-item scale completed by parents, measuring children’s eating behaviour using seven subscales; “Satiety Responsiveness/Slowness in Eating”, “Fussiness”, “Food Responsiveness”, “Enjoyment of food”, “Desire to Drink”, “Emotional Undereating”, and “Emotional Overeating” [25]. Overall, the scale had good internal consistency (*a* = 0.81), along with each sub-scale: “Satiety Responsiveness/Slowness in Eating” (*a* = 0.61), “Fussiness” (*a* = 0.71), “Food Responsiveness” (*a* = 0.87), “Enjoyment of Food” (*a* = 0.87), “Desire to Drink” (*a* = 0.91), “Emotional Undereating” (*a* = 0.81) and “Emotional Overeating” (*a* = 0.83).

#### Procedure

Parents were invited to participate in the research via Prolific, and they were reimbursed for their time (£6.00/h). After completing the information sheet and consent forms, participants answered demographic questions about their child. They then went on to complete the MEBS-P and CEBQ. This was to see if children’s mindful eating behaviours predicted any other eating behaviours as expected. No children participated in this study. Following completion of both scales, participants read the debrief form.

#### Data analysis

Correlations were performed between the MEBS-P and CEBQ to determine associations between mindful eating behaviours and other eating behaviours. All data were analysed using IBM SPSS 30.

#### Results

There was a significant, moderately positive relationship between “Non-judgemental Awareness” and “Food Enjoyment” (*r* = 0.36, *p* = 0.4) amongst female participants. No other significant correlations were observed between the MEBS-P and CEBQ (Table 13).

**Table 13:** Pearson’s correlations were conducted between the MEBS-P and the CEBQ for female participants

	1	2
1. MEBS-P (NJA)	1	
2. CEBQ (FE)	0.363*	1

(1) MEBS-P (NJA) = *The Mindful Eating Behaviour Scale for Parents,* Non-judgemental Awareness, (2) CEBQ (FE) = *Children’s Eating Behaviour Questionnaire,* Food Enjoyment

^*^Correlation is significant at the 0.05

## Discussion

Addressing limitations in previous research, the present study aimed to develop and validate new psychometric scales for children and parents, to measure mindful eating behaviour in children. Study 1 produced strong ratings from expert participants regarding the content validity of the scale, showing the scale was appropriate for children, aligned with mindful eating behaviour, and was suitable for further testing. Study 2 showed the occurrence of two factors for both child and parent scales, supporting the original factorial structure of the MEBS-T that reflected the two components of mindful eating; that is, *sensory attention* and *non-judgmental awareness* [17]. All items strongly loaded onto each factor, supporting that the scale had good internal consistency. In Study 3, both scales showed strong correlations between the two testing points over the two-week interval, indicating high test–retest reliability. The results in studies 1–3 were consistent with previous research as this scale was based on an existing, validated scale for adults using the precise definition for mindful eating behaviour [17].

Study 4 explored the criterion-related validity of the scale by examining its relationships to existing measures of children’s eating behaviours and mindfulness. “Sensory Attention” was correlated with “Restrained Eating”, and “Non-judgemental Awareness” was correlated with “Conformity Motive”. These are novel findings that suggest that higher levels of mindful eating behaviour in children are associated with higher dietary restraint and eating under social influences. After observing mostly non-significant associations in the full sample, the analysis reverted to an exploratory investigation and controlled gender to explore potential individual differences and enhance the understanding of the data. The results showed that girls who reported higher mindfulness also showed higher levels of mindful eating behaviour. This is theoretically consistent as there would be an expectation for mindfulness and mindful eating to significantly correlate. It is also consistent with previous research showing greater improvements in eating behaviours (e.g. increased fruit consumption, reduced emotional eating) and increases in mindful eating in girls compared to boys following mindfulness-based interventions, suggesting that girls may be more responsive to mindful approaches [12, 21, 35]. In girls, the “Non-judgemental Awareness” subscale also correlated with higher levels of mindfulness, indicating that this scale may be a good predictor of mindful behaviour in children.

Interestingly, the present study found that higher levels of mindful eating behaviour were associated with higher overall K-PEMS scores in boys. Significant relationships were observed between the MEBS-C and subscales with “Emotional Eating” and “Coping”, “Social”, and “Reward Enhancement” motives to eat palatable foods. This suggests that boys who eat more mindfully may still eat emotionally as well as consume low-nutritional-value foods for reasons other than physiological hunger. These findings contrast with previous research, which suggests that boys who eat more mindfully are less likely to engage in emotional or mindless eating [12, 36], but findings need to consider that the scale used in the present research is substantially different to previous interpretations of mindful eating.

Study 5 findings showed an association between “Food Enjoyment” and “Non-judgemental Awareness", suggesting that girls who eat mindfully enjoy their food more. This is a novel finding and contrasts with previous findings that reported mindfulness strategies do not improve food enjoyment in children [13] but also supports how mindful eating influences food enjoyment in adults [37, 38]. The original validation of the CEBQ suggested non-differential satiety cues and slowness in eating, proposing that some of the elements are not captured in younger samples of children [25]. Future research should focus on utilising other eating behaviour scales or replicate this research with an older sample of children.

An important consideration that should be made is that the parental version cannot capture children’s internal awareness or subjective experiences during eating, and are prone to be influenced by parental beliefs, personal beliefs, societal expectations and self-perceptions. Conversely, the child version may be limited by developmental constraints on introspection and recall. The differences reflect informant‑specific perspectives rather than measurement shortcomings, the correlation between parent and child versions offers only construct level validity, not as measurement equivalence, and the parallel child and parent measures offer a theoretically grounded, developmentally sensitive approach to assessing mindful eating.

There are several limitations that need to be considered. First, children and parents may give responses that are not entirely accurate, increasing the risk of social desirability bias. Parents may also influence their child’s responses when they are present during data collection, which was the case for the present studies. Parents may guide their child’s answer by correcting them or reacting in a way that leads to the child answering differently to what they would have. If a parent already holds strong beliefs around food and eating behaviour, the child may feel pressure to answer that align with their expectations [39]. As the scale is a self-report measure designed to assess children’s own behaviours and attitudes towards food, parent intervention may impact the self-report nature of it. Future research should explore uncensored responses and potentially explore control protocols for social desirability.

Second, parental demographic information was not collected for any of the studies, reason being that the focus of the research was specifically on children’s eating behaviours. However, collecting data on parental demographics and parental eating behaviours could assist for controlling variables that could influence children’s eating behaviours. Future research using the scales should consider collecting data on parent demographics and eating behaviours.

Third, although the scale displays strong suitability for children, it is possible that some children may struggle to fully understand the questions. The way questions are interpreted by the children may be an element that is further affecting the validity of the scale, and is worth exploring without any parental guidance, potentially through a think-aloud study [40].

Fourth, the majority of the participants were of a White British background, and other ethnic groups were minimal. Previous studies have shown that different cultures show different inclinations to children’s eating behaviours and parental feeding practices [41]. For example, research shows that Black Afro-Caribbean parents demonstrate high levels of restrictive and controlling feeding, particularly when the child is of a heavier weight, compared to White-German and White-British parents [41]. Research also shows that Hispanic parents perceive mindful eating as food appreciation, family time, enjoying the sensory experiences of food, whereas non-Hispanic parents use mindful eating to focus on hunger cues and portion control [42]. These are two very different approaches to mindful eating, further supporting that perceptions of eating behaviours vary across cultures and ethnicities. Therefore, the limited ethnic diversity of the current sample may limit the generalisability of the findings to children from other cultural backgrounds. Such limitation in not surprising as participants were recruited via Prolific, which predominantly samples from WEIRD (Western, Educated, Industrialised, Rich, and Democratic) populations. Prolific samples are appropriate for the initial scale validation, providing sufficient heterogeneity and data quality, so that the present research can evaluate dimensionality, reliability, and construct relationships, but broader population and cultural validation can be addressed in subsequent work. Future research should aim to include more ethnically and culturally diverse populations to assess the cross-cultural validity of these findings.

Fifth, the “Sensory Attention” subscale produced a Cronbach’s alpha that was slightly shy from the acceptable value of 0.7, and the elimination of item 2 marginally skipped the scale at 0.7 + for both studies 2 and 4. Then again, the use in analyses did not signify any differences between the 3- and 4-item subscale, and there is literature that suggests that the a Cronbach’s α value of 0.50 or higher is considered satisfactory when scales are short and have fewer items [43]. Item 4 on the “Sensory Attention” subscale of the MEBS-P “my child fully tastes every bite that they are eating” is potentially ambiguous. The phrase “every bite” is particularly problematic, as many parents may be reluctant to endorse it even if their child is generally attentive to sensory experiences. It is therefore recommended that future research consider removing the phrase “every bite”. Additionally, to estimate internal consistency, McDonald’s omega (ω) may be a better recommendation for future research, as unlike Cronbach’s alpha, calculations are not dependent on item numbers, and scales are not automatically penalised for having fewer items. Indeed, the problems observed with Cronbach’s alpha were not observed when McDonald’s omega was used. Future research may need to make similar decisions on retaining item 2 or using McDonald’s omega.

Sixth, the criterion-related validity studies produced conflicting results with previous research [12, 13]. However, this is a new scale in a child population where there is currently limited research to compare findings, and the scale developed in this research is based on a theory that did not conflate mindful eating with other eating behaviours. Study 4 and study 5 both aimed to examine how mindful eating behaviour can predict other eating behaviours in children, however there were limited associations. Although findings suggest a small indication that mindful eating behaviours can influence other eating behaviours, for example, food enjoyment in girls, directions for future research are to investigate different eating behaviour scales as well as differences in eating motives between boys and girls. Finally, while EFA is appropriate for identifying the latent structure, the absence of a confirmatory factor analysis (CFA) using an independent sample is required to formally test and confirm the stability and generalisability of the proposed factor model. As such, conclusions regarding the factor structure should be viewed as preliminary, and future research should prioritise CFA.

In conclusion, the development and validation of the MEBS-C and MEBS-P provide valuable and precise tools for advancing future research on childhood eating behaviours. By accurately aligning with the established definition of mindful eating behaviour, the MEBS-C and MEBS-P address a critical gap in existing literature, offering unparallel accuracy in examining children’s mindful eating. Importantly, its dual use will offer researchers the opportunity to expand the existing literature by assessment and comparison of general, parent and child contexts of eating behaviours; ultimately leading to the development or refinement of more effective mindful eating interventions. The present manuscript presents foundational work that promises to promote healthier, more enjoyable eating experiences for children and families, and contribute significantly to a healthier future child population.

### Strengths and limits

The present research presents the following limitations: potential social desirability bias, possible misunderstandings of items from children, limited ethnic diversity and marginal internal consistency on the “*Sensory Attention*” subscale. Criterion-related validity results were also inconsistent with previous research. Strengths are the validation of two new Mindful Eating Behaviour Scales for children and parents.

### What is already known on this subject?

Mindful eating is a strategy that can assist children and parents in promoting healthy eating. Existing definitions have led to current psychometric measurements lacking validity and alignment to mindful eating behaviour.

### What this study adds?

The study introduced novel measurement tools to assess mindful eating behaviours in children, through two versions, the Child or Parent version of the Mindful Eating Behaviour Scale, by addressing previous research methodological issues and aligning with the definition of mindful eating. This research offers reliable and valid assessments for individuals, researchers, and practitioners. The findings have implications for both research and interventions by addressing gaps in the existing literature and advancing theoretical understanding of children’s mindful eating behaviours.

## Data Availability

Data will be available on request from the corresponding author.

## Appendix A

### The Mindful Eating Behaviour Scale for Children (MEBS-C)

Here are some things people say about eating. Please tell me how true each one is for you when you are eating.

**Table:**

	I really disagree (1)	I kind of disagree (2)	I kind of agree (3)	I really agree (4)
1. I really taste and enjoy my food	○	○	○	○
2. I pay attention to how my food smells, feels, and looks	○	○	○	○
3. I focus on what I’m eating	○	○	○	○
4. I make sure to taste every bite of my food	○	○	○	○
5. When I have thoughts and feelings that have nothing to do with eating, I bring my attention back to the food and eating	○	○	○	○
6. When I'm eating and I have thoughts and feelings, I stay focused on the food	○	○	○	○
7. I stay focused on my food even when other thoughts and feelings pop up	○	○	○	○
8. I think about eating and how good the food is and do not spend time with other thoughts and feelings	○	○	○	○

Add all 8 items for an overall score on Mindful Eating Behaviour. Items 1–4 constitute the Sensory Attention subscale, and items 5–8 constitute the Non-judgmental Awareness subscale. Please mix the items in a random order to avoid repetitive items.

These questions would be suitable for children aged 8–12 years old.

## Appendix B

### The Mindful Eating Behaviour Scale for Parents (MEBS-P)

There is a list of statements below. Please use the rating scale to indicate how well each statement describes your child's experience with eating and food.

**Table:**

	Strongly Disagree (1)	Disagree (2)	Agree (3)	Strongly Agree (4)
1. My child fully tastes what they are eating	○	○	○	○
2. My child notices the smell, texture, and/or colors of the food they are eating	○	○	○	○
3. My child focuses on what they are eating	○	○	○	○
4. My child fully tastes every bite that they are eating	○	○	○	○
5. My child notices thoughts and/or feelings that are unrelated to their eating but redirects their attention to the food and the experience of eating	○	○	○	○
6. When my child is eating, they have thoughts and/or feelings but keep refocusing on the food	○	○	○	○
7. My child holds their attention on what they are eating despite recognizing the occurrence of thoughts and/or feelings while eating	○	○	○	○
8. When my child is eating, they overcome unrelated thoughts and/or feelings by focusing on the food and the sensation of eating	○	○	○	○

Add all 8 items for an overall score on Mindful Eating Behaviour. Items 1–4 constitute the Sensory Attention subscale, and items 5-8 constitute the Non-judgmental Awareness subscale.
